# Functional kleptoplasts intermediate incorporation of carbon and nitrogen in cells of the Sacoglossa sea slug *Elysia viridis*

**DOI:** 10.1038/s41598-020-66909-7

**Published:** 2020-06-29

**Authors:** Sónia Cruz, Charlotte LeKieffre, Paulo Cartaxana, Cédric Hubas, Najet Thiney, Sofie Jakobsen, Stéphane Escrig, Bruno Jesus, Michael Kühl, Ricardo Calado, Anders Meibom

**Affiliations:** 1 ECOMARE, CESAM - Centre for Environmental and Marine Studies, Department of Biology, University of Aveiro, Campus Universitário de Santiago, 3810-193 Aveiro, Portugal; 2 UMR CNRS 6112 LPG-BIAF, Université d’Angers, 2 Boulevard Lavoisier, 49045 Angers, Cedex 1 France; 3 Biologie des Organismes et Ecosystèmes Aquatiques (BOREA), Muséum National d’Histoire Naturelle, Sorbonne Université, Université de Caen Normandie, Université des Antilles, CNRS, IRD, Station Marine de Concarneau, Place de la croix, 29900 Concarneau, France; 4 Marine Biological Section, Department of Biology, University of Copenhagen, 3000 Helsingør, Denmark; 5 Laboratory for Biological Geochemistry, École Polytechnique Fédérale de Lausanne, 1015 Lausanne, Switzerland; 6 Laboratoire Mer Molécules Santé, Faculté des Sciences et des Techniques, Université de Nantes, 44322 Nantes, France; 7 Center for Advanced Surface Analysis, Institute of Earth Sciences, University of Lausanne, 1015 Lausanne, Switzerland; 8 Present Address: Cell & Plant Physiology Laboratory, University of Grenoble Alpes, CNRS, CEA, INRA, Grenoble, France

**Keywords:** Ecology, Physiology, Plant sciences, Zoology

## Abstract

Some sacoglossan sea slugs incorporate intracellular functional algal chloroplasts, a process termed kleptoplasty. “Stolen” chloroplasts (kleptoplasts) can remain photosynthetically active up to several months, contributing to animal nutrition. Whether this contribution occurs by means of translocation of photosynthesis-derived metabolites from functional kleptoplasts to the animal host or by simple digestion of such organelles remains controversial. Imaging of ^13^C and ^15^N assimilation over a 12-h incubation period of *Elysia viridis* sea slugs showed a light-dependent incorporation of carbon and nitrogen, observed first in digestive tubules and followed by a rapid accumulation into chloroplast-free organs. Furthermore, this work revealed the presence of ^13^C-labeled long-chain fatty acids (FA) typical of marine invertebrates, such as arachidonic (20:4*n*-6) and adrenic (22:4*n*-6) acids. The time frame and level of ^13^C- and ^15^N-labeling in chloroplast-free organs indicate that photosynthesis-derived primary metabolites were made available to the host through functional kleptoplasts. The presence of specific ^13^C-labeled long-chain FA, absent from *E. viridis* algal food, indicates animal based-elongation using kleptoplast-derived FA precursors. Finally, carbon and nitrogen were incorporated in organs and tissues involved in reproductive functions (albumin gland and gonadal follicles), implying a putative role of kleptoplast photosynthesis in the reproductive fitness of the animal host.

## Introduction

Kleptoplasty is the capacity of a non-photosynthetic host to retain functional chloroplasts from algal sources (therefore termed kleptoplasts or kleptochloroplasts)^1^. It was first identified by Kawaguti and Yamasu^2^ in the marine slug *Elysia atroviridis* and since then, the presence of functional algal chloroplasts was reported in several other species of sacoglossan molluscs^3–7^ as well as in other organisms, such as ciliates^8^, foraminiferans^8,9^ or dinoflagellates^10^, and more recently in the flatworms *Baicalellia solaris* and *Pogaina paranygulgus*^11^. However, sacoglossan sea slugs remain the only known metazoans in which kleptoplasts remain functional for several months^7^. Such long-term maintenance of kleptoplasts photosynthetic activity in sacoglossan sea slugs is puzzling, as the algal nucleus has been digested and most of the genetic machinery playing a role in plastid regulation has been transferred to the nucleus over the evolution of endosymbiosis^12^. The importance of kleptoplasty for the nutrition and metabolism of sacoglossan sea slugs remains controversial. Most studies have shown that autotrophy plays an important role in individual survival and fitness^3,13,14^, but it has also been argued that photosynthesis may not be crucial for the survival of sacoglossan sea slugs^15^. While the translocation of photosynthesis-derived products from a viable autotrophic unicellular symbiont to its host is well established^16,17^, such translocation from viable kleptoplasts to the sea slug host is still debated^13,15,18,19^. Earlier radiolabeled carbon-based studies suggested translocation of photosynthates into sacoglossan sea slugs cells^20,21^. In *Tridachia crispata* and *Tridachiellia diomedea*, ^14^C radioactivity was detected within 2 h in what was assumed to be the renopericardium, the cephalic neural tissue and the mucus secreting pedal gland^21^. The detection of radioactivity in kleptoplast-free organs at such sort-time frame was indicative of incorporation via functional kleptoplasts in these two species. In another species, *Elysia timida*, it was recently proposed that, after some days of starvation, starch accumulated at the kleptoplasts and could be used by the animal after kleptoplast digestion^18,19^. Another potential benefit mediated by kleptoplasty that remains unexplored in photosynthetic sea slugs is nitrogen assimilation. While kleptoplasts can provide carbon substrates to the host, short periods of starvation could rapidly lead to nitrogen deficiency, unless kleptoplasts could somehow mediate nitrogen acquisition^22^. Indeed, previous observations of uptake of ^15^N-labeled ammonium, nitrite and urea (but not nitrate) into bulk tissues and specific amino acids enrichment of *Elysia viridis* hosting functional kleptoplasts, suggests a light-dependent assimilation of nitrogen in this organism^22^.

This work presents new evidence for light-dependent incorporation of inorganic carbon and nitrogen into tissues of the sacoglossan sea slug *Elysia viridis*, using compound specific isotope analysis (CSIA) of fatty acid methyl esters (FAME) coupled with high-resolution secondary ion mass spectrometry (NanoSIMS), demonstrating spatial and temporal movements of ^13^C and ^15^N isotopes.

## Results

Isotopic dual labeling pulse experiments were conducted with *E. viridis* individuals incubated for 12 h with ^13^C-bicarbonate and ^15^N-ammonium. Different animals were sampled at increasing time points up to 12 h of labeled inorganic carbon and nitrogen addition (see Materials and Methods for details).

### Microscopy observations

**Figure 1:**
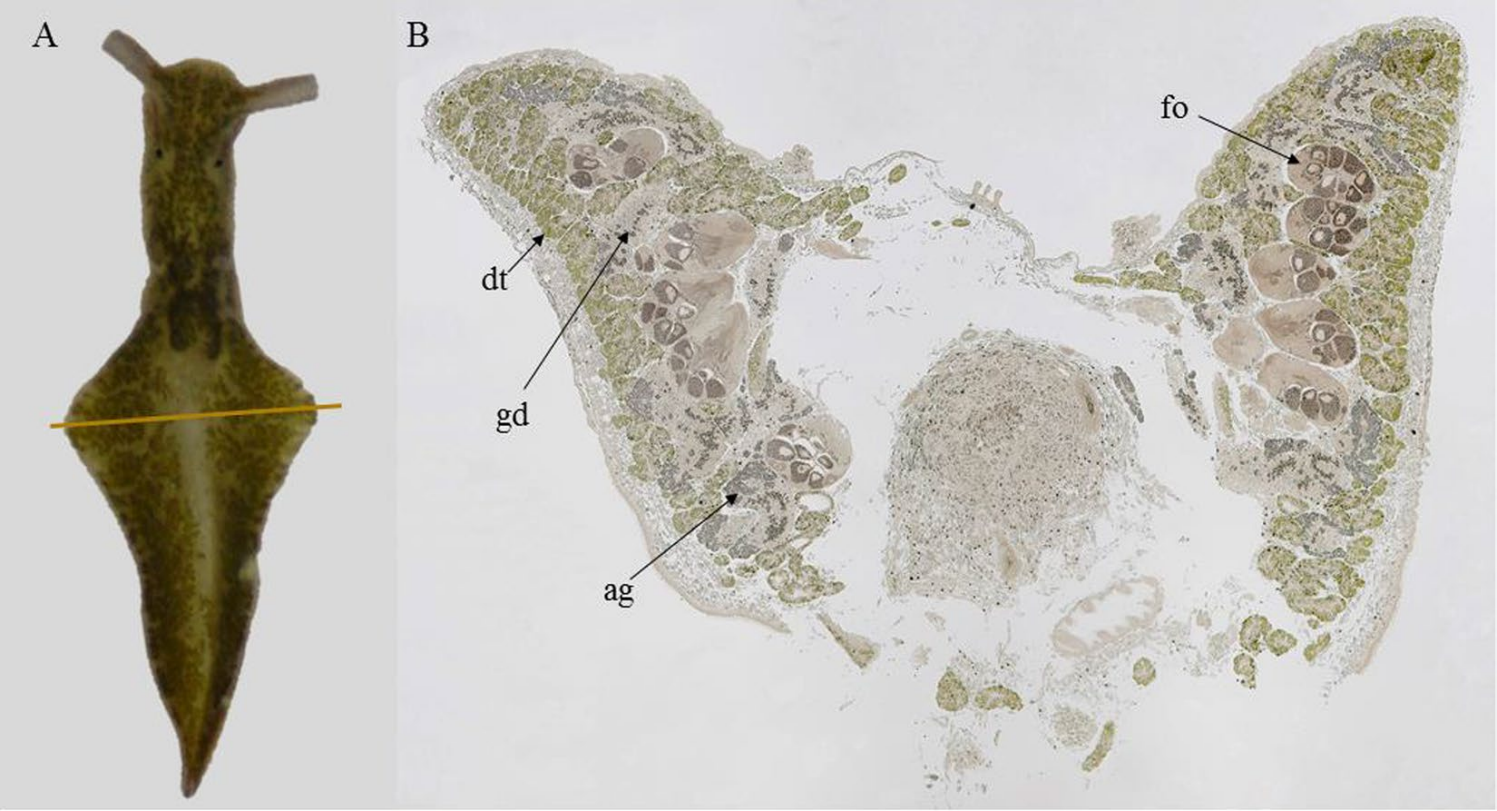
Sacoglossan sea slug *Elysia viridis*. (**A**) Whole individual showing ramified digestive tubules throughout the body, brown line indicates the sectioning region for further microscopy and imaging analysis of carbon and nitrogen assimilation; (**B**) Representative transversal overview section immediately after the pericardium region, as indicated in (**A**). No staining was applied. ag: albumin gland, dt: digestive tubule, gd: gland duct, fo: gonadal follicle.

**Figure 2:**
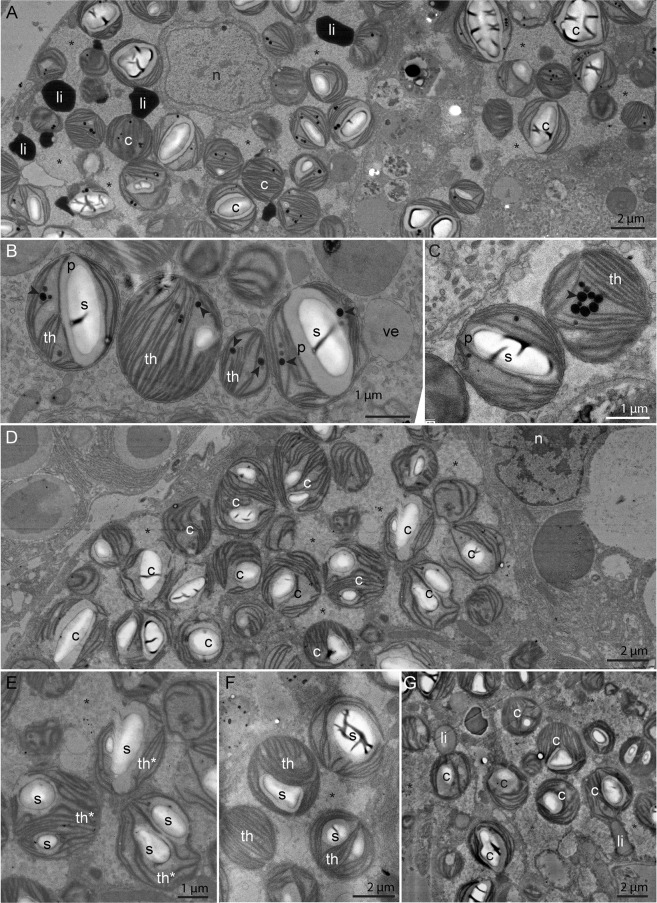
Transmission Electron Microscopy (TEM) micrographs of the ultrastructure of sacoglossan sea slugs *Elysia viridis* cytoplasm and its kleptoplasts. (**A**) Kleptoplasts in the digestive tubule after 1.5 h of incubation. (**B**,**C**) Detailed structure of the kleptoplasts from specimens incubated for 1.5 h (**D**) Kleptoplasts in the digestive tubule after 12 h of incubation. (**E**,**F**) Detailed structure of the kleptoplasts from specimens incubated for 12 h. (**G**) Kleptoplasts and lipid droplets association in the digestive tubule after 12 h of incubation. Arrowheads: plastoglobuli; asterisks: flaky electron-lucent cytoplasm surrounding the kleptoplasts; c: chloroplast; li: lipid droplets; n: nucleus; p: pyrenoid; th: thylakoid; th*: loose thylakoid; s: starch; ve: vesicle.

Transversal sections of *E. viridis*, immediately after the pericardium region, allowed the visualization of digestive tubules, the albumin gland, gland ducts and gonadal follicles (Fig. 1). Histological identification was based on anatomical works of *E. viridis* and other sacoglossan sea slugs^23–27^. Electron microscopy observations of the digestive tubules revealed intact sequestered kleptoplasts after 1.5 h of incubation (Fig. 2a), with clearly distinguishable ultrastructural features, such as thylakoids, starch grains, pyrenoids and plastoglobuli (Fig. 2b,c). Kleptoplasts were equally abundant in the digestive tubules after 12 h of incubation (Fig. 2d). While some of the kleptoplasts were intact, others had more diffuse thylakoids and membranes (Fig. 2e,f). All kleptoplasts observed in sea slugs incubated for 12 h lacked plastoglobuli but had intact starch grains. After both 1.5 and 12 h of incubation, kleptoplasts were surrounded by flaky electron-lucent cytoplasm (Fig. 2, indicated by asterisks).

Numerous lipid droplets could be seen in the digestive tubules of the sea slugs incubated for 1.5 h (Fig. 2a), while lipid droplets were scarce in two of the three sea slugs incubated for 12 h (Fig. 2d). In one of the three sea slugs incubated for 12 h, a few lipid droplets were observed in the digestive tubules, and some of them were in close association with the kleptoplast membranes (Fig. 2g).

### ^13^C-labeled fatty acid composition

**Figure 3:**
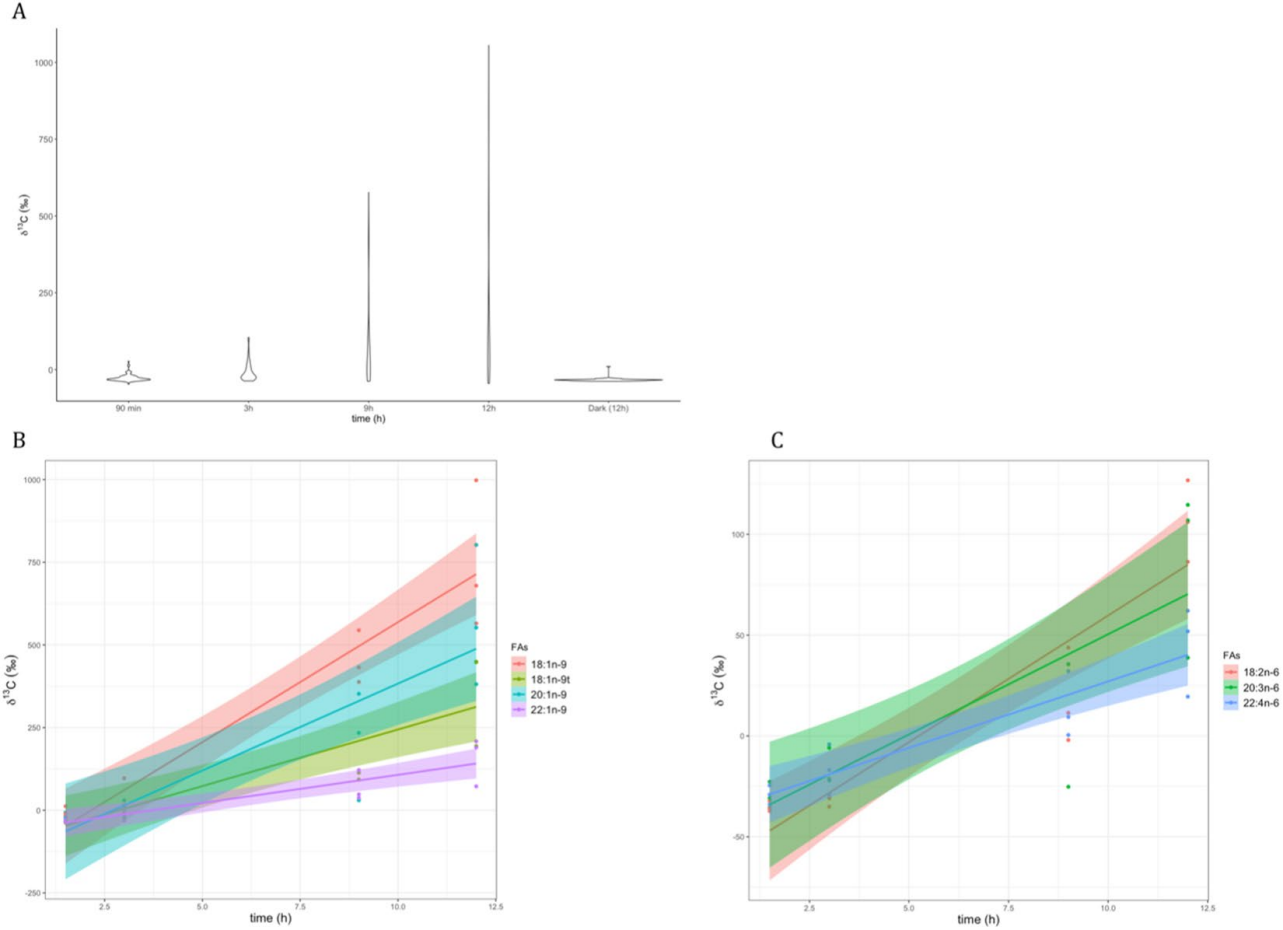
Incorporation of ^13^C into fatty acids (FAs) of sacoglossan sea slugs *Elysia viridis* incubated for 1.5, 3, 9 and 12 h in artificial seawater enriched with 2 mM NaH^13^CO_3_ and 20 µM ^15^NH_4_Cl in the presence of light. (**A**) Sum of δ^13^C of all FA fractions at each time point. (**B**) n-6 polyunsaturated FA (PUFAs) and (**C**) n-9 monounsaturated FA (MUFAs) families along the incubation periods. See Table S2 for FAs δ^13^C-averaged-values.

The most abundant fatty acids observed in *E. viridis* were the saturated 16:0 and 18:0, the monounsaturated (MUFAs) 18:1*n*-9 and 20:1*n*-9 and the polyunsaturated (PUFAs) 18:3*n*-3, 20:4*n*-6, 20:5*n*-3 and 22:4*n*-6 (Table S1). In the presence of light, individuals incubated in ^13^C-bicarbonate enriched ASW for 1.5, 3, 9 and 12 h showed an increasing incorporation of ^13^C into FAs over time (Fig. 3). Incorporation of ^13^C occurred in some of the most abundant FAs in *E. viridis*, such as 20:1*n*-9 (gondoic acid) and the 22:4*n*-6 (adrenic acid) (Table S2). When kept under dark conditions during 12 h of incubation with ^13^C-bicarbonate enriched ASW, sea slugs showed incorporation of ^13^C equivalent to that of conspecifics incubated in the presence of light but in non-enriched ASW (Table S2). Incorporation of ^13^C into polyunsaturated *n*-6 (PUFAs 18:2*n*-6, 20:3*n*-6 and 20:4*n*-6) and monounsaturated *n*-9 (MUFAs 18:1*n*-9, 20:1*n*-9 and 22:1*n*-9) FAs is highlighted in Fig. 3b,c. The slope of carbon isotopic ratios over time was higher in FAs such as 18:2*n*-6 and 18:1*n*-9,and lower in longer-chain FAs such as 22:4*n*-6 and 22:1*n*-9.

### Imaging of ^13^C and ^15^N assimilation

**Figure 4:**
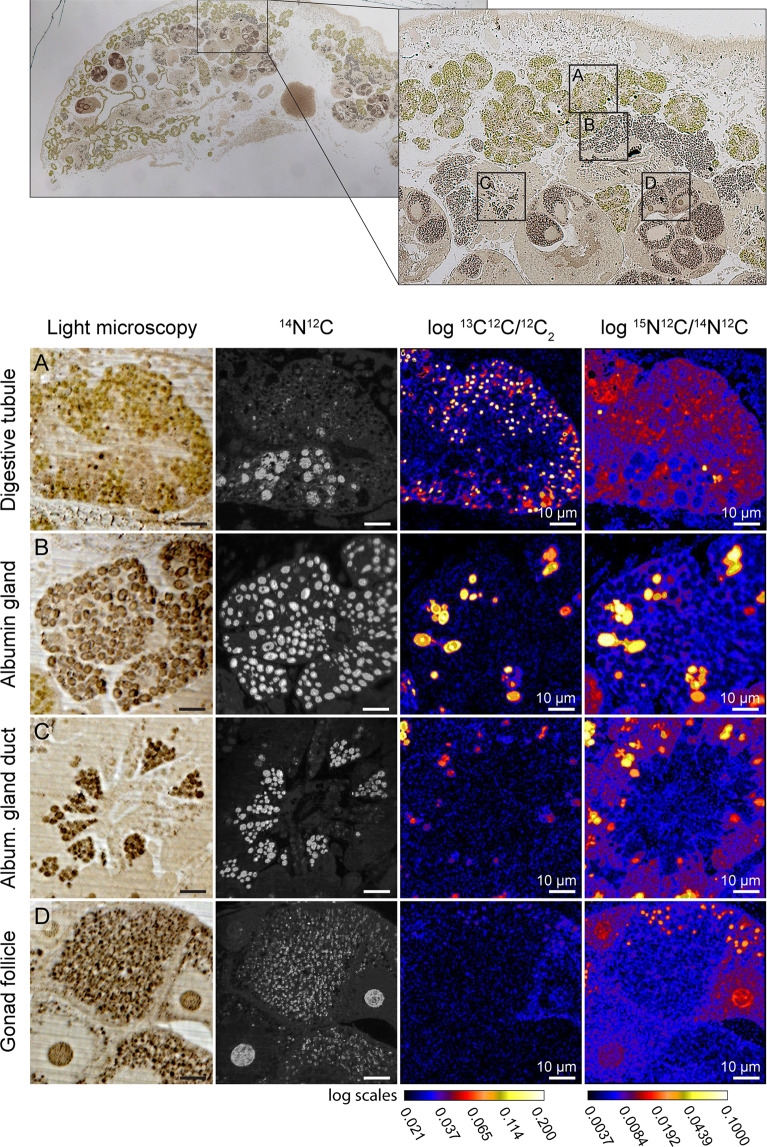
Light microscopy and complementary NanoSIMS images of a sacoglossan sea slug *Elysia viridis* incubated for 12 h in artificial seawater enriched with 2 mM NaH^13^CO_3_ and 20 µM ^15^NH_4_Cl in the presence of light. (Top) Light microscopy images highlighting areas of interest imaged with NanoSIMS. (**A**–**D**) ^13^C and ^15^N enrichment in different organs of *E. viridis*: digestive tubule, the albumin gland, the gland duct and the gonadal follicles. Time-evolution (1.5, 3, 6 and 12 h) of the ^13^C and ^15^N assimilation in the different organs of *E. viridis* can be seen in Fig. 5 (albumin gland) and Supplementary Fig. S1 (digestive tubule), S2 (gland duct) and S3 (gonadal follicles).

**Figure 5:**
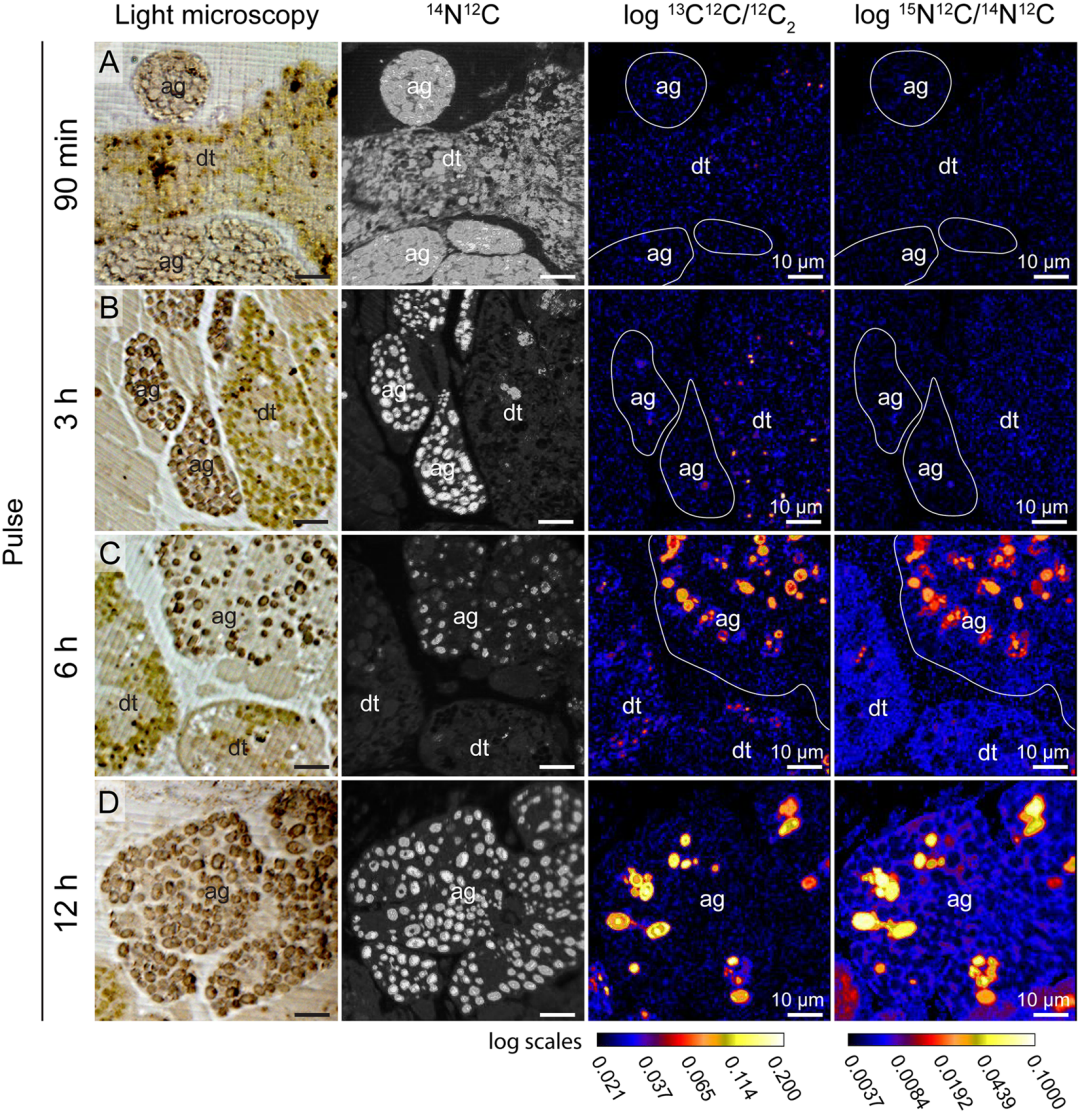
Time-evolution of the ^13^C and ^15^N assimilation in the albumin glands of *Elysia viridis*. Light microscopy pictures and complementary NanoSIMS images of the sea slug incubated in artificial seawater enriched with 2 mM H^13^CO_3_ and 20 µM ^15^NH_4_, in the presence of light, for (**A**) 1.5 h, (**B**) 3 h, (**C**) 6 h and (**D**) 12 h. ag: albumin gland, dt: digestive tubule. Time-evolution of the ^13^C and ^15^N assimilation in other related organs of *E. viridis* can be seen in supplementary Fig. S1 (digestive tubule), S2 (gland duct) and S3 (gonadal follicles).

All NanoSIMS ^13^C/^12^C and ^15^N/^14^N images from the individuals incubated for 12 h in the light with ^13^C-bicarbonate and ^15^N-ammonium showed ^13^C and ^15^N-labeling in all the observed tissues, i.e., digestive tubules (Fig. 4a), albumin gland (Fig. 4b) and gland ducts (Fig. 4c), and gonadal follicles (Fig. 4d). All tissues exhibited similar enrichment dynamics; overall they increased from low-enrichment after 1.5 h of incubation to higher enrichment values after 12 h of incubation (Figs. 5 and S1–S3). It was clear from observations of semi-thin sections, combined with NanoSIMS imaging, that ^13^C- and ^15^N-labeling was not homogenously distributed in the different tissues, instead ^13^C and ^15^N-hotspots could be identified in all images.

All individuals incubated in the dark for 12 h with ^13^C-bicarbonate and ^15^N-ammonium displayed no ^13^C-enrichments (Fig. S4). In contrast, ^15^N-labeling was observed in all sea slug tissues (i.e., digestive tubules, gland ducts, albumin gland and follicles), albeit at a much lower level than in conspecifics incubated under light (compare Figs. 4 and S4).

**Figure 6:**
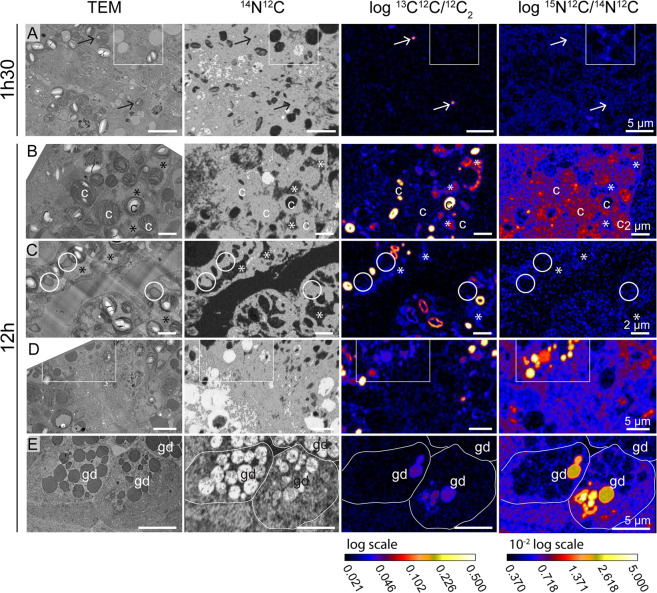
Transmission Electron Microscopy (TEM) micrographs and complementary NanoSIMS images of different isotopically labeled structures at different time of incubations in artificial seawater enriched with 2 mM NaH^13^CO_3_ and 20 µM ^15^NH_4_Cl in the presence of light. (**A**) Digestive tubule after 1.5 h of incubation. Arrows are pointing at early ^13^C-labeled starch in some kleptoplasts. (**B**) Kleptoplasts in the digestive tubule after 12 h of incubation. (**C**) Kleptoplasts and lipid droplets (circles) in the digestive tubule after 12 h of incubation. (**D**) Dense circular structures and vesicles in the digestive tubule after 12 h of incubation. (**E**) Gland duct after 12 h of incubation. Higher magnification TEM micrographs of the isotopically labeled areas surrounding by squares can be seen in the Supplementary Figs. S5 and S6. Asterisks: flaky electron-lucent cytoplasm surrounding the kleptoplasts; c: chloroplast; gd: gland duct.

Electron microscopy observations combined with NanoSIMS imaging of individuals incubated in the light for 1.5 and 12 h allowed the visualization of specific isotopically labeled organelles. In the digestive tubules, after 1.5 h of incubation, only the starch of some kleptoplasts was ^13^C-labeled (Figs. 6a and S5). Of the three specimens incubated for 1.5 h, only one exhibited low ^15^N-labeling in some unidentified vesicles and secretory vesicles associated with the Golgi apparatus (Fig. S6). After 12 h of incubation, the three specimens analyzed were labeled with ^13^C and ^15^N. In the digestive tubules, ^13^C-labeling was mainly concentrated in the kleptoplast starch and/or pyrenoids, but was also spread in the cytoplasm with a flaky aspect surrounding the photosynthetic organelles (Figs. 6b,c and S5). The lipid droplets observed in one of the specimens in close association with the kleptoplasts membranes were not labeled (Figs. 6c and S5). After 12 h, ^15^N-labeling was mainly concentrated in the thylakoids of the kleptoplasts and also spread in the cytoplasm of the digestive tubule, with high variability being recorded among specimens (Figs. 6b,c and S6). In the digestive tubules, a few unidentified vesicles, as well as some unidentified dark circular structures with a diameter of ca. 2 µm, were also ^15^N-labeled (Figs. 6d and S5). Finally, some of the dark vesicles on the gland duct were also labeled, both in ^13^C and ^15^N (Fig. 6e).

## Discussion

### Carbon assimilation

*De novo* synthesis of algal FAs occurs in chloroplasts and results in the production of saturated FAs (SFA), typically 16:0, from acetyl-CoA^28^. These precursor SFAs are then modified through a series of elongation and desaturation steps to produce various unsaturated FAs. Conversion from 18:1*n*-9 to 18:2*n*-6 and 18:3*n*-3 requires Δ12 and Δ15 desaturase enzymes, which are found in primary producers^29^. The PUFAs 18:3*n*-3 (ALA), 18:2*n*-6 (linoleic acid) and 20:3*n*-6 (DELA) are typically produced by green macroalgae^30^, while longer chain FAs with higher levels of unsaturation are more commonly present in animals^30,31^. The SFA 16:0 and the PUFAs 18:2*n*-6, 18:3*n*-3 were among the most abundant FAs found in *E. viridis*, likely originating from its dietary algal source *Codium tomentosum*^32^. On the contrary, the longer MUFAs and PUFAs found abundantly in *E. viridis*, such as 20:1*n*-9, 20:2*n*-6, 20:2*n*-9, 20:4*n*-6, 22:2*n*-9, 22:4*n*-6 and 22:5*n*-3, are not present in *C. tomentosum*^32^. Therefore, the presence of both arachidonic acid 20:4*n*-6 and adrenic acid 22:4*n*-6 in the sea slug in such a short time frame between ^13^C-incubation and the detection of labelled metabolites (12 h), can only be explained by an elongation process mediated by the animal host using algal *n*-6 FA precursors. Such elongation pathway has already been reported for other marine invertebrates^33^. Similarly, the FA 22:5*n*-3, which was abundant in *E. viridis*, can also be produced from its precursor 20:5*n*-3^33^, the latter being present in *C. tomentosum*^32^ but displaying a much higher relative abundance in *E. viridis* (respectively, ca. 2.5% vs. 7.5%). The *n*-9 MUFAs and PUFAs 20:1*n*-9, 20:2*n*-9 and 22:2*n*-9 are present in an elongation pathway known to occur in marine mollusks^34^. Indeed, these non-methylene-interrupted dienoics (NMID) C20 and C22 FAs are known to be synthesized by mollusks through a Δ5 desaturase enzyme acting upon the appropriate precursor, 18:1*n*-9, to promote elongation^31,34^.

Chloroplasts are rich in 18:2*n*-6, which can be transformed in arachidonic acid (20:4*n*-6) only trough a series of desaturation and elongation steps. It is interesting to note that ^13^C incorporation rate (as shown by the slope of the regression curves in Fig. 3) is higher for 18:2*n*-6 than 20:4*n*-6, indicating that the carbon donor (Acetyl CoA→Malonyl-CoA) during the elongation process is not ^13^C-enriched and thereby not provided by the chloroplast but rather by the host. However, it is impossible to tell if algal 18:2*n*-6 was obtained by the host trough chloroplast degradation (likely from Diacylglycerols or phospholipids) or trough lipid transport^35^. We therefore hypothesize the following path explaining the presence of labeled long-chain FAs which have not been identified in the algal food source: 1) *de novo* synthesis of FAs at the kleptoplast level, 2) transfer of algal FAs to the animal cell, and 3) recycling in the animal cell (e.g. elongation/desaturation). Considering the short time-frame in which these three processes occurred and the fact that within 12 h there is no obvious kleptoplast digestion in TEM observations (Fig. 2) we further hypothesize that the step (2) on the path described above is due to translocation from intact kleptoplasts, rather than digestion. This hypothesis is further supported by the fact that digestion of kleptoplasts in a different species, *E. timida*, occurred only after prolonged starvation (at least 21 days according to Laetz *et al*.^18^) as detailed in the following paragraphs.

It was recently shown that kleptoplasts in fed *Elysia timida* retained only small amounts of starch, while when deprived from their food source (starved), kleptoplasts in *E. timida* started to accumulate starch^18^. The starting point of starch accumulation was variable, occurring after 3, 10 or 21 days of starvation (first stage of starvation condition), depending on the group being tested (different locations and/or different seasons were tested). After further periods of starvation (second stage), starch degradation was higher than starch production. Again the time point of this switch was variable and depended of the targeted group, occurring after 21 and 42 to 88 days. Moreover, it was also coincident with the decline in photosynthetic activity. This suggests that, in a first stage starch accumulated while kleptoplasts are mostly functional, while during a second stage of long starvation kleptoplasts were degraded and starch digestion occurred^18^, either by the host and/or by the kleptoplasts to meet their metabolic needs. It is therefore assumed that kleptoplasts are being degraded/digested after long periods (days to weeks) of starvation. Although similar information does not exist for *E. viridis*, this species has been shown to retain functional kleptoplasts for at least 3 months^3^, so this species could display a similar pattern to that described for *E. timida*. In the present work, a significantly shorter time frame was used, with TEM-NanoSIMS data showing ^13^C-labeled intact starch grains in the kleptoplasts after 12 h of incubation under light (Figs. 3 and 6). Furthermore, after 3 h hours, ^13^C-labeling was already observed in different chloroplast-free organs such as the albumin gland (Fig. 5). These observations of a very fast ^13^C-translocation to distant animal tissues strongly support an active transfer of photosynthates from the kleptoplasts to host tissues, rather than a ^13^C-incorporation by digestion of kleptoplast starch grains. Nevertheless, it cannot be ruled out, that sea slugs with long-term retention of functional kleptoplasts may also acquire C-compounds derived from photosynthates from the digestion of older kleptoplasts, as hypothesized previously^15,18^. Sacoglossan sea slugs may thus adopt different strategies when chloroplasts-replenishment is impaired for long periods of time, with both pathways being concomitant or occurring at different time scales.

The translocation of photosynthetically acquired carbon to animal tissues was previously hypothesized for two other species of sacoglossan sea slugs, *Elysia* (=*Tridachia*) *crispata* and *Tridachiella diomedea*^21^. Despite the lower resolution histological approach to pinpoint the presence of ^14^C in these organisms, these authors suggested that after 15 min of incubation only chloroplast-containing tissues, i.e., digestive tubules, were labeled. Subsequently, ^14^C-incorporation was also detected in chloroplast-free organs, such as the renopericardium (after 60 min), the cephalic neural tissue and the mucus secreting pedal gland (after 120 min), and the intestine (after 300-540 min). In *T. diomedea*, ^14^C was still tracked in animal tissues after 6 weeks of food deprivation. In *E. viridis*, we first observed ^13^C-labeling after 1.5 h in starch grains of kleptoplasts present in digestive tubules (Fig. 6). After longer incubation times, ^13^C-labeling was detected in all animal tissues surveyed: albumin gland, gland ducts and gonadal follicles (Figs. 4–6 and S1–S3). Therefore, inorganic ^13^C was first photosynthetically accumulated into kleptoplasts starch, followed by a C-transfer to sea slug tissues, conceivably by translocation of soluble C-compounds (sugars, organic acids) or fatty acids. In the present work, the absence of ^13^C-labeling in lipid droplets does not support the hypothesis of a direct transfer via lipid droplets across kleptoplast membranes, as seen in algae or suggested for kleptoplastidic foraminifera and the sacoglossan *Elysia chlorotica*^17,36,37^. Instead, the ^13^C-labeled electron-lucent cytoplasm surrounding the kleptoplasts could correspond to FAs being transferred and reprocessed in the vicinity of kleptoplasts. Plastoglobuli, lipoprotein particles surrounded by a membrane lipid monolayer, here present in early stages of incubation (Fig. 2a–c), may also be a vessel for photosynthates transport between chloroplasts and the cytoplasm^38^.

### Ammonium assimilation

**Figure 7:**
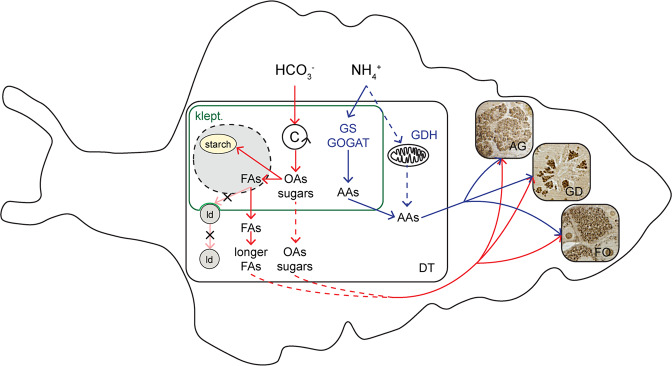
Schematic diagram of assimilation pathways of the inorganic carbon and ammonium in the sacoglossan sea slug *Elysia viridis*. Plain lines represent pathways demonstrated by the present study. Plain lines with a cross represent pathways suggested by the literature but that were shown to not occur in the present study. Dotted lines represent pathways that might occur in carbon or ammonium assimilation based on this work and from literature, but that could not be assessed with certainty. The grey area surrounded by a dotted black line represent compounds that could be assimilated by *E. viridis* on longer time-scale in the case of kleptoplast degradation/digestion. See details in the text. AAs: amino acids, AG: albumin gland, DT: digestive tubule, FAs: fatty acids, FO: gonadal follicle, GD: gland duct, GDH: glutamate dehydrogenase, GS/GOGAT: glutamine synthetase/glutamate synthase, klept.: kleptoplast, ld: lipid droplet, OAs: organic acids.

A pathway for ammonium assimilation by *E. viridis* feeding on the algae *Codium fragile* was suggested from incubations of sea slugs with ^15^N-labeled nitrogen substrates^22^, showing that part of ammonium assimilation was light-dependent, and decreased significantly when sea slugs were incubated with specific inhibitors of the GS and GOGAT kleptoplast enzymes. Thus, at least part of ammonium assimilation would have taken place through kleptoplastidic GS-GOGAT activity, acknowledging the possibility of an additional cytoplasmic pathway through the GDH enzyme. This enzyme was shown to occur in some marine mollusks^39,40^. Here, using an imaging approach we directly show ^15^N-assimilation in *E. viridis* previously fed with the algae *C. tomentosum*. A schematic diagram of the inorganic carbon and ammonium assimilation pathways in *E. viridis* is presented in Fig. 7, where plain lines represent pathways demonstrated by the present study. While no ^13^C-enrichment was recorded in FAs or in isotopic images of dark-incubated individuals, some dark-incubated ^15^N-assimilation was observed via NanoSIMS imaging. However, when incubated in the presence of light, sea slugs showed a much higher level of ^15^N-enrichment in their tissues. This light-dependent activity of kleptoplastidic enzymes might be explained by the fact that photosynthesis provides energy and carbon skeletons necessary for amino acid synthesis in the chloroplast. An external supply of nitrogen would be essential for processes such as *de novo* synthesis of plastid-encoded proteins, shown to occur in *Elysia chlorotica*^5^, a photosynthetic sacoglossan sea slug with the ability to retain functional kleptoplasts for extended periods of time (>6 months). Ammonium assimilation recorded in specimens incubated in the dark (albeit significantly reduced) could result either (i) from a cytoplasmic enzymatic pathway (GDH) of the sea slug itself, or (ii) from residual chloroplast activity. As for ^13^C-assimilation, we clearly show that the ^15^N-metabolites derived from ^15^N-ammonium assimilation were also found in the kleptoplasts and digestive tubules cytoplasm, as well as in kleptoplast-free tissues: albumin gland, gland ducts and gonadal follicles (Figs. 4 and 6). TEM-NanoSIMS analysis showed high ^15^N-labeling in some unidentified vesicles and in vesicles associated with the Golgi apparatus, thus identified as secretory vesicles (Fig. S6). Golgi apparatus are involved in the maturation of proteins, receiving these molecules from the endoplasmic reticulum and sending them to their next destination through secretory vesicles^41^.

## Conclusions

We show with unprecedented spatial and temporal resolution the incorporation of C and N in animal cells mediated by functional kleptoplasts. The present imaging of ^13^C- and ^15^N-enriched organs and tissues involved in reproductive functions (albumin gland and gonadal follicles) incite further research on the importance of photosynthesis for metabolic pathways involved in parental investment and offspring fitness in kleptoplastidic sea slugs. Future studies are required to confirm in other photosynthetic sacoglossan sea slugs the extent to which photosynthesis-derived primary metabolites are made available to the host from functional kleptoplasts.

## Methods

### Experimental design

The objective of the present study was to determine if the incorporation of inorganic carbon and nitrogen in the sacoglossan sea slug *Elysia viridis* was dependent on kleptoplast activity. Sea slugs were collected, acclimated to lab conditions, and incubated with and without isotopically labeled bicarbonate and ammonium as described bellow. The fate of carbon and nitrogen was analysed using compound specific isotope analysis (CSIA) of fatty acid methyl esters (FAME) coupled with high-resolution secondary ion mass spectrometry (NanoSIMS).

### Collection and acclimation

*Elysia viridis* specimens were collected on the intertidal rocky shore of Praia de Labruge (41°16′28.9″N; 8°43′45.3″W), Vila do Conde (Portugal) from their macroalgal food source *Codium tomentosum*. Specimens and respective food source were randomly distributed between two 10 L tanks filled with filtered natural seawater, at salinity 35, 16 °C and under a photon irradiance (400-700 nm) of 30 µmol photons m^–2^ s^–1^ at the water surface level (14 h:10 h light:dark cycle) for two weeks acclimation before isotopic labeling experiments.

### Dual isotopic labeling incubations

Isotopic dual labeling pulse experiments were conducted in closed-systems (1 L glass bottles, 3 independent containers per treatment). Enriched artificial seawater (ASW) was made in accordance with Harrison *et al*.^42^ but using NaH^13^CO_3_ (^13^C isotopic abundance of 99%, SIGMA-ALDRICH) and ^15^NH_4_Cl (^15^N isotopic abundance of 98%, SIGMA-ALDRICH) to a final concentration of 2 mM and 20 µM, respectively (enriched-ASW). Control non-enriched-ASW contained NaH^12^CO_3_ and ^14^NH_4_Cl in the same concentrations as enriched-ASW. The pulse of isotopic dual labeling started 30 min after the onset of the light period. Sacoglossan sea slugs *E. viridis* with a length between 9 and 12 mm were incubated in the absence of their food source both under an incident photon irradiance of 80 µmol photons m^–2^ s^–1^ (400-700 nm; white fluorescence lamps) and in dark conditions. Dark conditions served as a control for bicarbonate and ammonium assimilation that could potentially be incorporated into animal tissue in a light-independent manner via exchange reactions and/or carboxylation steps in animal metabolism. Individuals in enriched-ASW and exposed to light were sampled after 1.5, 3, 9 and 12 h (n = 3), quickly rinsed with distilled water, immediately frozen in liquid nitrogen and stored at –80 °C for further fatty acids (FAs) analysis. Another subset of three individuals at 1.5, 3, 6 and 12 h were collected, rinsed and fixed in 0.2 M cacodylate buffer containing 4% glutaraldehyde and 0.5 M sucrose and stored at 4 °C for 24 h before tissue preparation for further microscopy and secondary ion mass spectrometry (SIMS) imaging analysis (NanoSIMS 50 L). Additional subsets of three individuals from non-enriched-ASW exposed to light and from enriched-ASW kept in darkness (control treatments) were sampled after a 12 h incubation period and frozen or fixed, as described above, for FAs and SIMS analysis.

### Fatty acid composition

Fatty acids (FAs) were extracted following the method described by Meziane *et al*.^43^ Lipids were extracted by sonication (35 kHz, 20 min) using chloroform/methanol/water (2:1:1,v:v:v). Complex lipids such as triglycerides or phospholipids were hydrolized by saponification (90 min, 90 °C) with NaOH:MeOH (1:2, v-v) to release individual FAs. An internal standard (tricosanoic acid: 23:0, 10 μg) was added to each sample before extraction. Individual FAs were then derivatized into fatty acids methyl esters (FAMEs) using boron-trifluoride methanol (BF_3_-MeOH) at 90 °C for 10 min. Samples were then dried under N_2_ flux and transferred to hexane for injection in a gas chromatograph (GC, VARIAN CP-3800 equipped with flame ionization detector - FID). Most FAMEs were identified by comparing their retention times with those of known standards (SUPELCO 37, PUFA-1 Marine Source, and Bacterial Mix; SUPELCO Inc., Bellefonte, PA, United States). FAMEs were further identified by GC coupled to a mass spectrometer (GC-MS, VARIAN GC450-220MS). For both devices, FAMEs separation was performed using a SUPELCO OMEGAWAX 320 column (30 m × 0.32 mm i.d., 0.25 μm film thickness) with He as carrier gas. After injection of 1 μl of sample at 60 °C, the temperature was raised to 150 °C at 40 °C min^–1^, then to 240 °C (held 14 min) at 3 °C min^–1^. FAMEs were systematically corrected for the added methyl group and corresponding individual FAs are designated in this study as CX:Y-nZ, where X is the number of carbons, Y the number of double bonds and Z the position of the ultimate double bond from the terminal methyl.

### Compound specific isotope analysis (CSIA) of fatty acid methyl ester (FAME)

After GC and GC-MS analyses, carbon stable isotopic ratios (expressed in ‰) of individual fatty acids were measured by gas-chromatography-isotope ratio mass spectrometry (GC-IRMS). Measurements were performed at the UC Davis Stable Isotope Facility of the University of California (Davis, CA, United States). FAMEs dissolved in hexane were injected in splitless mode and separated on a VARIAN factorFOUR VF-5ms column (30 m × 0.25 mm ID, 0.25 micron film thickness). Once separated, FAMEs were quantitatively converted to CO_2_ in an oxidation reactor at 950 °C. Following water removal through a nafion dryer, CO_2_ enters the IRMS. δ^13^C values were corrected using working standards composed of several FAMEs calibrated against NIST standard reference materials. Stable carbon isotope ratios for individual FA were calculated from FAME data by correcting for the one carbon atom in the methyl group that was added during the derivatization process. This correction was made according to Gladyshev *et al*.^44^ by taking into account the isotope ratio of the derivatized methanol (BF_3_-methanol, –39.1‰ in our study), and the fractional carbon contribution of the free FA to the ester.

1 $${{\rm{\delta }}}^{13}{{\rm{C}}}_{{\rm{FA}}}(\textperthousand )=({{\rm{\delta }}}^{13}{{\rm{C}}}_{{\rm{FAME}}}-(1\,-{\rm{f}})\,\cdot \,{{\rm{\delta }}}^{13}{{\rm{C}}}_{{\rm{CH}}3{\rm{OH}}})/{\rm{f}}$$

where δ^13^C_FA_ is the isotopic composition of the free FAs, δ^13^C_FAME_ is the isotopic composition of the FA methyl ester, f is the fractional carbon contribution of the free FA to the ester and δ^13^C_CH3OH_ is the isotopic composition of the methanol derivatization reagent. The isotopic composition of the methanol was determined by the same GC-IRMS system.

### Tissue preparation for microscopy and NanoSIMS imaging

Sea slugs kept in the fixative for 24 h at 4 °C were transferred to 0.2 M cacodylate buffer with decreasing sucrose concentrations (15 min in cacodylate buffer with 0.5 M sucrose, then 0.25 M sucrose and finally no sucrose) and finally transferred to 2% osmium in distilled water for 1 h at room temperature in the dark. Sea slugs were then dehydrated in an increasing series of ethanol concentrations (two times 10 min in 50, 70 and 95% ethanol and four times 10 min in 100% ethanol; room temperature) followed by two times 10 min in acetone before resin embedding. Sea slugs were transferred to acetone:epon resin (1:1) overnight before being fully embedded in 100% epon resin for 6 h in a turning wheel. Sea slugs were finally transferred to new 100% epon resin, stored 30 min at room temperature for air bubbles removal, then 48 h at 60 °C for drying.

### Microscopy

Overview semi-thin cuts of 1.5 µm thickness were made from the sea slug body part roughly after the pericardium. Semi-thin sections were cut on a Leica UC7 ultramicrotome using a LEICA glass knife and were placed on circular glass cover slips (AGAR SCIENTIFIC, borosilicate, 10 mm diameter, 1½ mm thickness). Histological overviews were documented on an optical light microscope. Thin sections (70 nm) for electron microscopy observations were made on a LEICA ULTRACUT S microtome with a diamond ultra knife (40°) at the electron microscopy facilities of Angers University (SCIAM). The thin-sections were transferred onto formvar-carbon film copper grids and stained for 10 min with uranyless prior observation. The sections were observed with a transmission electron microscope (TEM, Philips 301 CM100, 80 kV) at the electron microscopy facility of Lausanne University (EMF). TEM grids (thin-sections) were transferred on 10 mm aluminum disks with double stick Cu-tape. Before NanoSIMS analysis, both the thin and semi-thin sections were coated with a ca. 15 nm thick gold layer.

### NanoSIMS isotopic image acquisition and data processing

Large areas of interest covering a far-reaching portion of digestive tubules, albumin glands, gland ducts, and gonadal follicles were imaged with optical light microscopy or TEM, and were then analyzed with a NanoSIMS 50 L secondary ion mass spectrometer (Laboratory for Biological Geochemistry, EPFL, Lausanne, Switzerland). This allowed imaging and quantification of the subcellular distribution of ^13^C and ^15^N enrichment in the exact same areas of the imaged sea slugs tissue, enabling direct correlation of structural and isotopic images. All measurements were performed using the following analytical conditions: 16 keV primary ion beam of Cs^+^ focused to a beam spot of ca. 100–150 nm and counting ^12^C^12^C^−^, ^13^C^12^C^-^, ^14^N^12^C^-^ and ^15^N^12^C^-^ ions in electron multipliers at a mass resolution of> 8000 (Cameca definition), enough to resolve potential interferences in the mass spectrum. Images captured with NanoSIMS 50 L were processed using the L’IMAGE PV-WAVE software (version 10.1, Larry R Nittler, Carnegie Institution of Washington, Washington DC, USA, https://sites.google.com/carnegiescience.edu/limagesoftware/home). Regions of interest selecting individual anatomic structures were defined and ^13^C/^12^C and ^15^N/^14^N ratios distribution maps were obtained by taking the ratio between the drift-corrected ^13^C^12^C^−^ and ^12^C^12^C^−^ images, and ^15^N^12^C^−^ and ^14^N^12^C^−^ images, respectively. Five stacked planes were used for each image. ^13^C and ^15^N enrichment values in the figures were expressed as logarithmic values of the measured ratios, i.e., ^13^C^12^C/^12^C_2_ and ^15^N^12^C/^14^N^12^C. These ratios were also measured in the controls (natural values): ^13^C^12^C/^12^C_2_ was 0.0210 ± 0.0002, and ^15^N^12^C/^14^N^12^C was 0.0037 ± 0.0001.

## Supplementary information

Supplementary information.

Supplementary information 2.

Supplementary information 3.

Supplementary information 4.

Supplementary information 5.

Supplementary information 6.

Supplementary information 7.

Supplementary information 8.
