# Supplementary material for: Functional kleptoplasts intermediate incorporation of carbon and nitrogen in cells of the Sacoglossa sea slug Elysia viridis
Source: Sci Rep. 2020 Jun 29;10:10548. doi: 10.1038/s41598-020-66909-7 (PMC7324368; doi:10.1038/s41598-020-66909-7)
Supplement: Supplementary file 1 — Supplementary information. [file 41598_2020_66909_MOESM1_ESM.docx]

**Table S2. Incorporation of ^13^C into fatty acids of the sacoglossan sea slugs *Elysia viridis***. δ^13^C values of three individuals (mean ± standard deviation) independently incubated for 1.5, 3, 9 and 12 h in artificial seawater (ASW) enriched with 2 mM NaH^13^CO_3_ in the presence of white light. Additionally, three sea slugs were also independently incubated for 12 h in the same conditions using (i) enriched-ASW but in the absence of light (“12h Dark”) and (ii) non-enriched-ASW in the presence of light (“12h Control”). Asterisk (*) indicates FAs that eluted together.

| **Treatment**  **FAs** | **1.5 h** | **3 h** | **9 h** | **12 h** | **12 h**  **Dark** | **12 h Control** |
| --- | --- | --- | --- | --- | --- | --- |
| **12:0** | -27 ± 2 | -21 | - | -19 | -28 | -27 ± 0 |
| **13:0** | -32 ± 0 | -32 ± 0 | -32 ± 0 | -32 ± 0 | -32 ± 0 | -32 ± 0 |
| **14:0** | -3 ± 28 | 61 ± 24 | 448 | 488 ± 59 | -30 ± 1 | -30 ± 0 |
| **15:0** | -24 ± 6 | 26 ± 7 | 36 ± 55 | 74 ± 76 | -34 ± 0 | -33 ± 1 |
| **15:1** | - | -21 | - | - | - | - |
| **16:0** | -10 ± 23 | 48 ± 50 | 412 ± 81 | 733 ± 213 | -36 ± 0 | -34 ± 1 |
| **16:1*n*-9**  **16:1*n*-7 *** | -26 ± 6 | -6 ± 18 | 234 ± 175 | 243 ± 11 | -34 ± 0 | -31 ± 1 |
| **16:2*n*-4** | - | -18 | 127 | - | - | - |
| **16:3*n*-4** | -37 ± 0 | -36 ± 2 | -38 ± 0 | -39 ± 5 | -38 ± 1 | -36 |
| **17:0** | -22 ± 13 | 38 ± 18 | 108 ± 3 | 200 ± 104 | -33 ± 1 | -31 ± 0 |
| **18:0** | -20 ± 12 | 9 ± 37 | 128 ± 98 | 91 ± 36 | -17 ± 24 | -29 ± 1 |
| **18:1*n*-9c** | -10 ± 26 | 37 ± 59 | 482 ± 85 | 791 ± 237 | -37 ± 1 | -36 ± 0 |
| **18:1*n*-9t** | -24 ± 8 | 5 ± 23 | 111 ± 16 | 386 ± 155 | -33 ± 1 | -30 ± 1 |
| **18:2*n*-6** | -35 ± 3 | -32 ± 3 | 21 ± 25 | 115 ± 21 | -38 ± 0 | -36 ± 1 |
| **18:3*n*-3** | -34 ± 1 | -31 ± 3 | -12 ± 6 | 0 ± 14 | -36 ± 1 | -35 ± 1 |
| **18:3*n*-4** | - | - | 65 | - | - | - |
| **18:3*n*-6** | -38 ± 8 | -27 ± 8 | -20 ± 6 | 23 ± 32 | -35 ± 0 | -34 ± 0 |
| **18:4*n*-1** | - | - | 109 | - | - | - |
| **18:4*n*-4** | -31 ± 1 | -29 ± 2 | 0 | 21 ± 48 | -33 ± 1 | -31 ± 2 |
| **20:0** | -26 ± 4 | -12 ± 5 | 20 ± 56 | 145 ± 92 | -32 ± 1 | -31 ± 2 |
| **20:1*n-*11 20:1*n*-13 *** | -27 ± 5 | -14 ± 10 | 50 ± 96 | 169 ± 64 | -33 ± 1 | -30 ± 2 |
| **20:1*n*-9** | -21 ± 15 | 10 ± 25 | 218 ± 171 | 609 ± 222 | -35 ± 1 | -34 ± 1 |
| **20:2**  **21:0 *** | -33 ± 4 | -20 ± 7 | 26 ± 16 | 71 ± 32 | -36 ± 1 | -35 ± 1 |
| **20:3*n*-3** | -27 ± 7 | -11 ± 18 | 31 ± 47 | 124 ± 52 | -34 ± 0 | -33 ± 1 |
| **20:3*n*-6** | -29 ± 7 | -15 ± 10 | 7 ± 45 | 93 ± 44 | -37 ± 0 | -35 ± 1 |
| **20:4*n*-6** | -32 ± 3 | -27 ± 3 | -18 ± 6 | -9 ± 11 | -35 ± 1 | -32 ± 1 |
| **20:5*n*-3** | -31 ± 2 | -30 ± 3 | -19 ± 6 | -9 ± 12 | -33 ± 0 | -31 ± 1 |
| **22:0** | -33 | -25 | 43 | 120 | -32 | -33 ± 2 |
| **22:1*n-*9** | -27 ± 7 | -16 ± 18 | 74 ± 48 | 166 ± 77 | -34 ± 1 | -33 ± 1 |
| **22:4*n*-6** | -29 ± 5 | -16 ± 13 | 16 ± 17 | 48 ± 23 | -34 ± 1 | -31 ± 1 |
| **22:5*n*-6** | -31 ± 5 | -9 ± 16 | 65 | 63 | -35 ± 1 | -36 ± 6 |
| **23:0** | -29 ± 1 | -30 ± 0 | -31 ± 1 | -32 ± 4 | -30 ± 0 | -28 ± 0 |
| **24:1*n*-9** | -34 | -24 ± 5 | - | - | -31 ± 3 | -30 ± 4 |
