# Supplementary figures and images for: Functional kleptoplasts intermediate incorporation of carbon and nitrogen in cells of the Sacoglossa sea slug Elysia viridis
Source: Sci Rep. 2020 Jun 29;10:10548. doi: 10.1038/s41598-020-66909-7 (PMC7324368; doi:10.1038/s41598-020-66909-7)

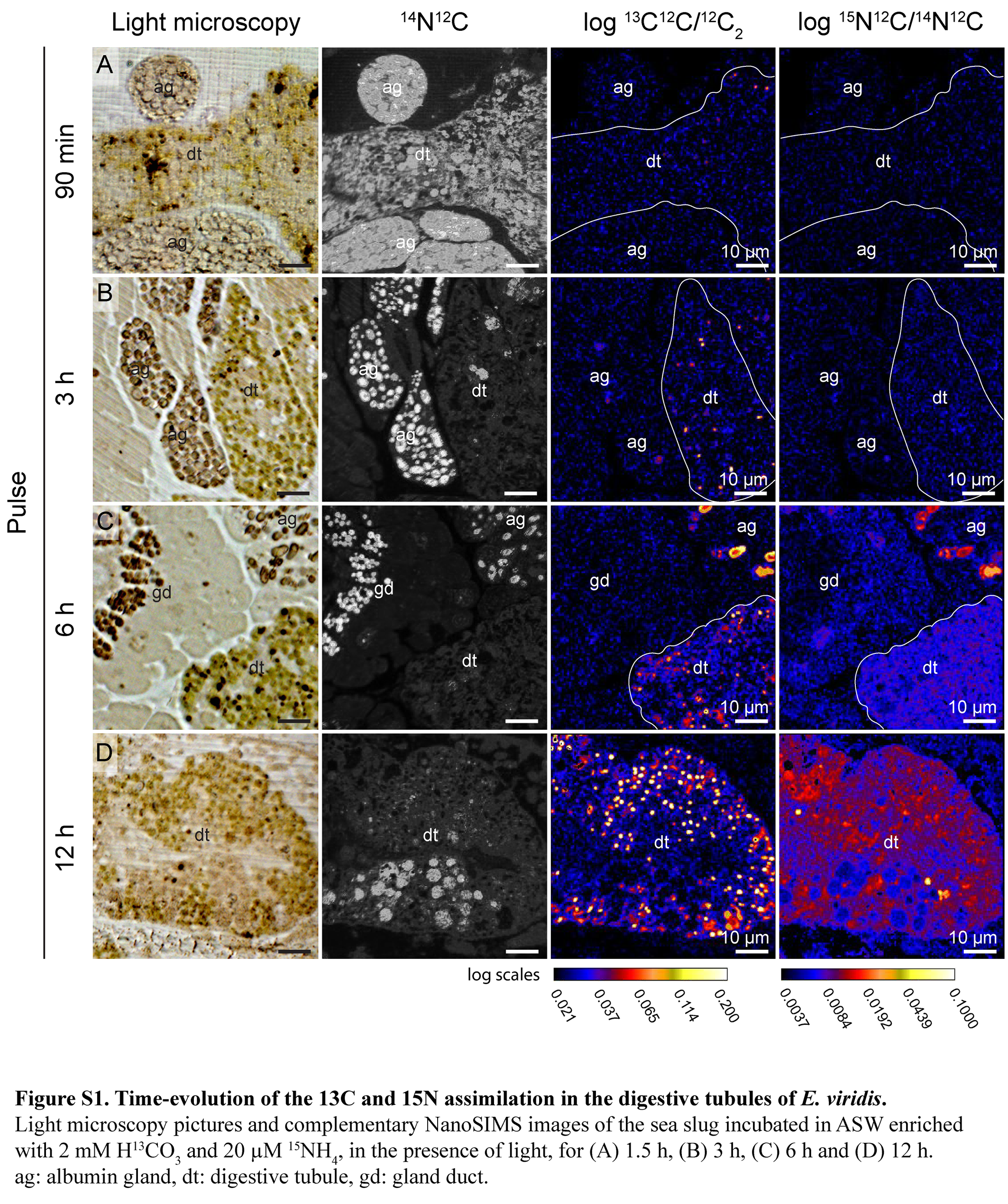

Supplement: Supplementary file 2 — Supplementary information 2. [file 41598_2020_66909_MOESM2_ESM.tif]

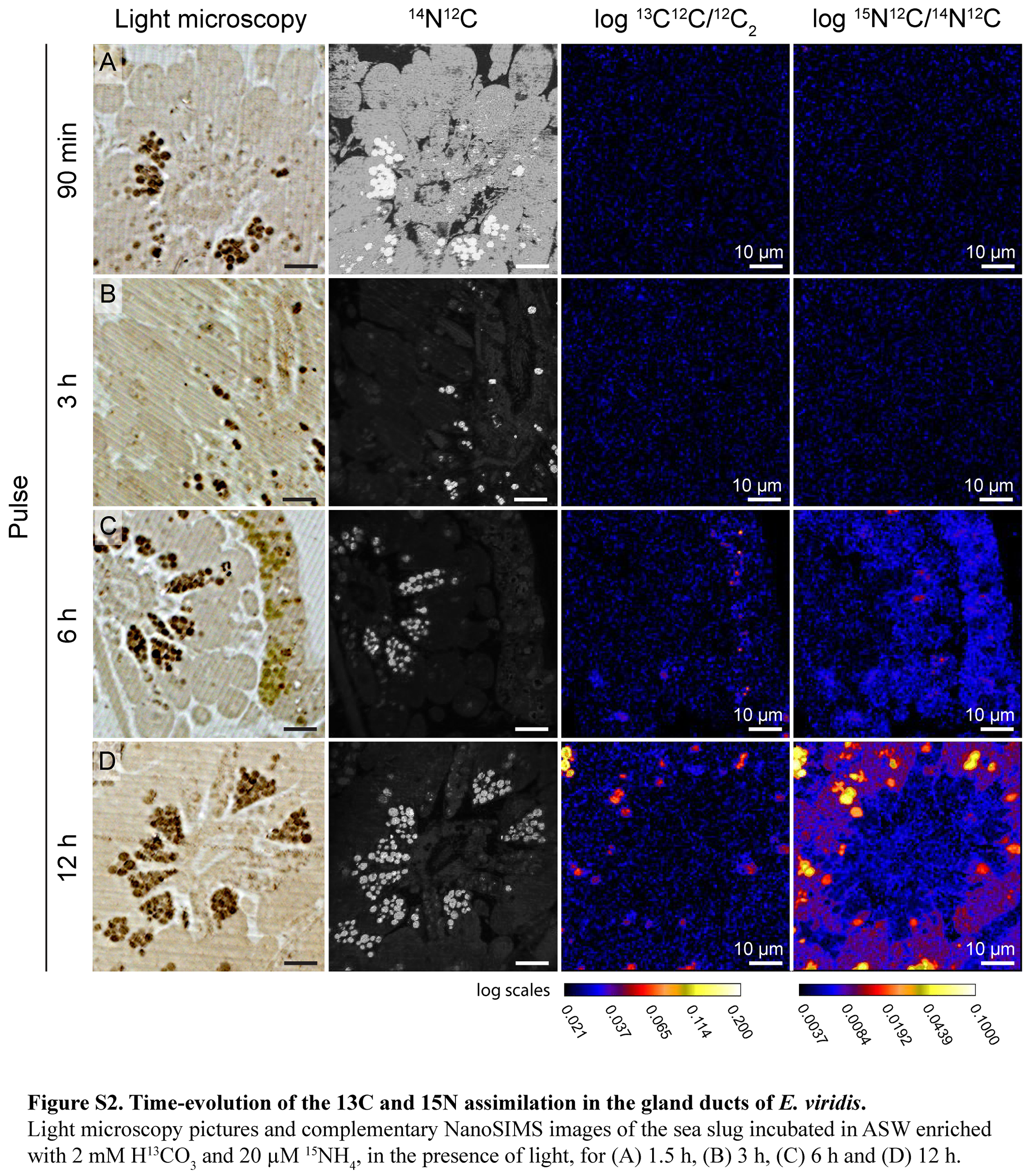

Supplement: Supplementary file 3 — Supplementary information 3. [file 41598_2020_66909_MOESM3_ESM.tif]

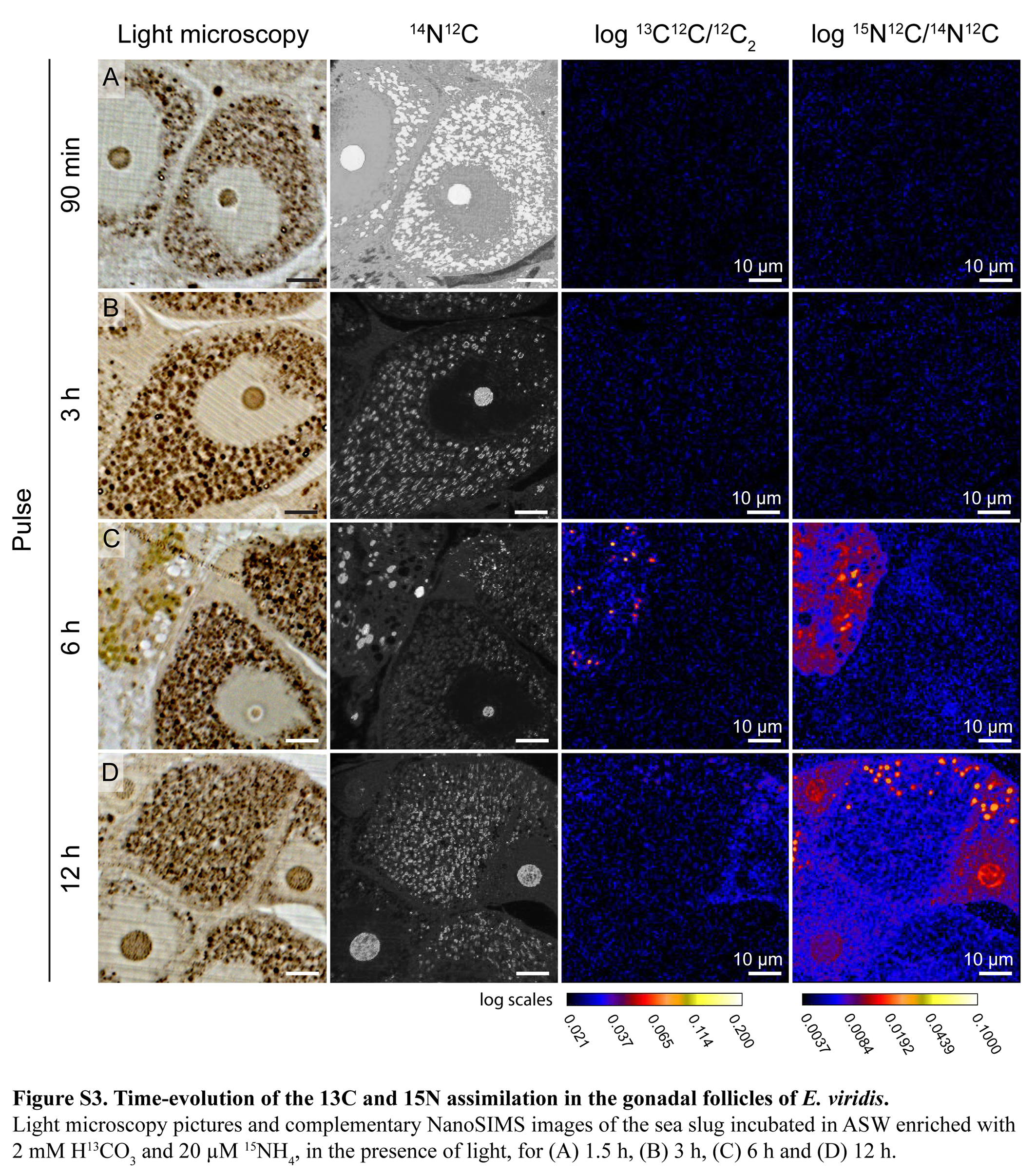

Supplement: Supplementary file 4 — Supplementary information 4. [file 41598_2020_66909_MOESM4_ESM.tif]

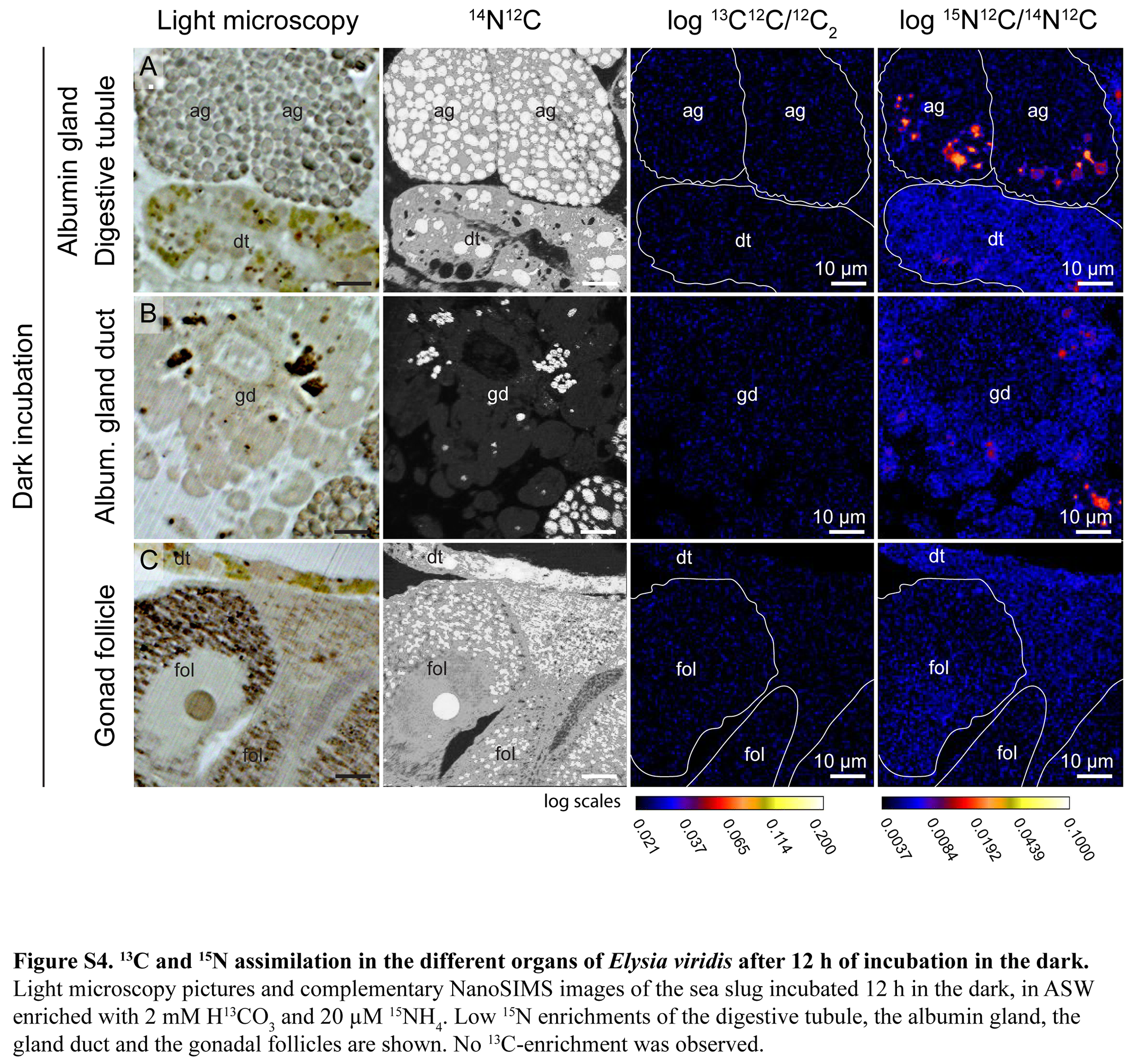

Supplement: Supplementary file 5 — Supplementary information 5. [file 41598_2020_66909_MOESM5_ESM.tif]

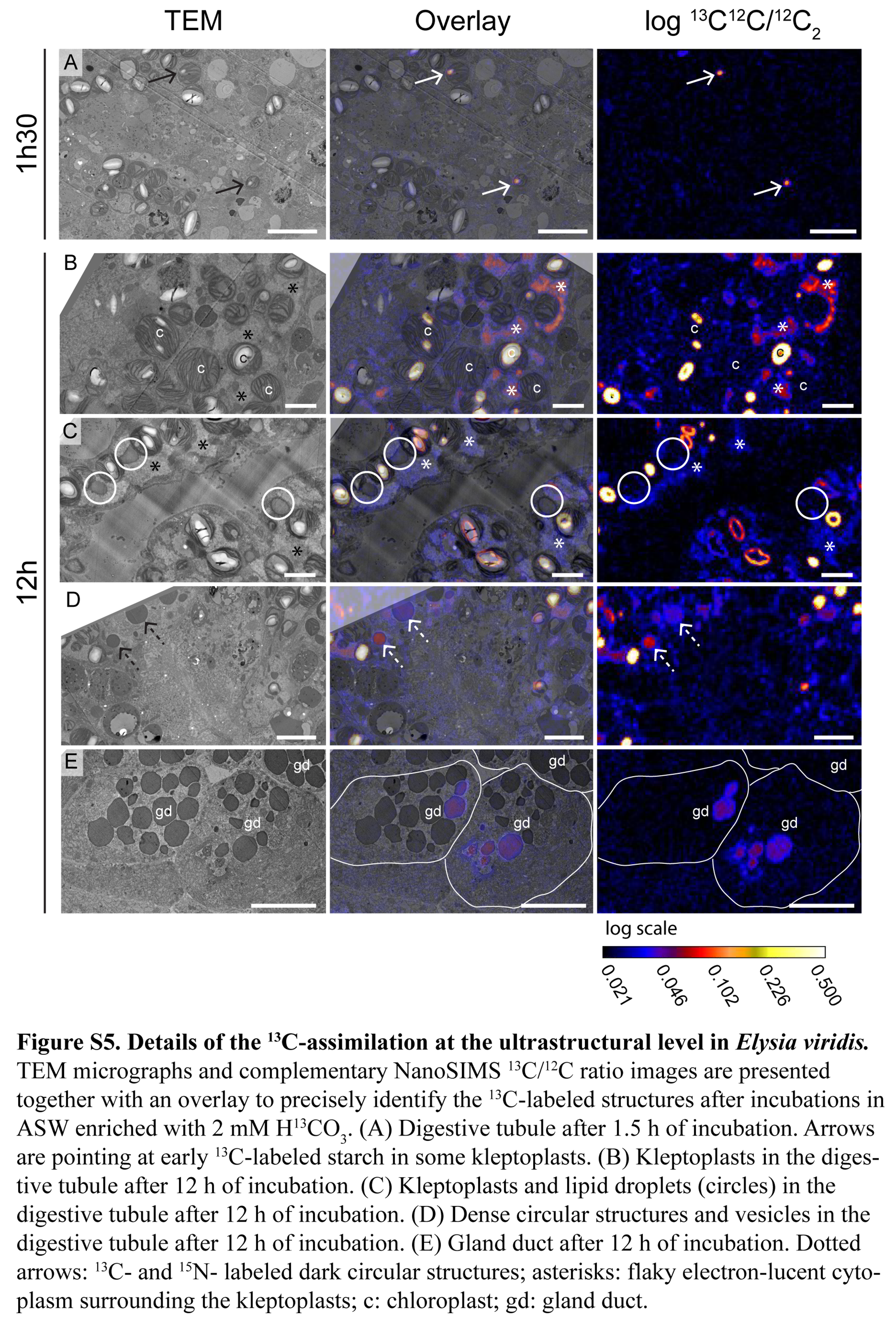

Supplement: Supplementary file 6 — Supplementary information 6. [file 41598_2020_66909_MOESM6_ESM.tif]

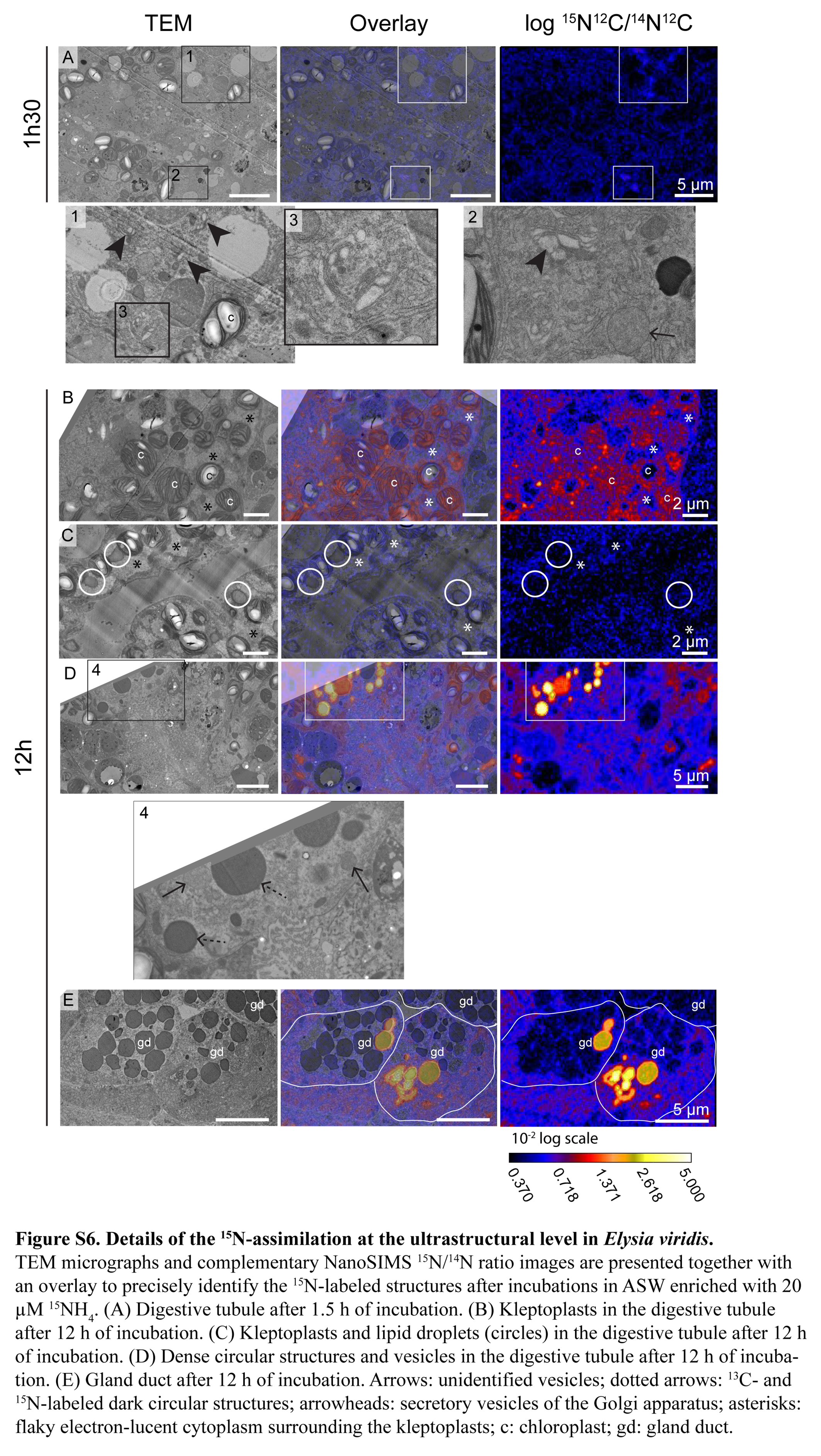

Supplement: Supplementary file 7 — Supplementary information 7. [file 41598_2020_66909_MOESM7_ESM.tif]
