# Supplementary material for: Functional kleptoplasts intermediate incorporation of carbon and nitrogen in cells of the Sacoglossa sea slug Elysia viridis
Source: Sci Rep. 2020 Jun 29;10:10548. doi: 10.1038/s41598-020-66909-7 (PMC7324368; doi:10.1038/s41598-020-66909-7)
Supplement: Supplementary file 8 — Supplementary information 8. [file 41598_2020_66909_MOESM8_ESM.docx]

**Table S1. Fatty acids (FA) relative abundances in the sacoglossan sea slugs *Elysia viridis***. Total relative abundances (%) from all FA identified in three individuals (mean ± standard deviation) independently incubated for 1.5, 3, 6, 9 and 12 h in artificial seawater (ASW) enriched with 2 mM NaH^13^CO_3_ in the presence of white light. Additionally, three sea slugs were also independently incubated for 12 h in the same conditions using enriched-ASW but in the absence of light (“12h Dark”) or non-enriched-ASW in the presence of light (“12h Control”).

| **Treatment**  **FAs** | **1.5 h** | **3 h** | **6 h** | **9 h** | **12 h** | **12 h**  **Dark** | **12 h**  **Control** |
| --- | --- | --- | --- | --- | --- | --- | --- |
| **12:0** | 0.4 ± 0.3 | 0.1 ± 0.0 | 0.1 ± 0.0 | 0.1 ± 0.0 | 0.5 ± 0.7 | 0.1 ± 0.1 | 0.2 ± 0.1 |
| **14:0** | 0.8 ± 0.1 | 0.7 ± 0.1 | 0.6 ± 0.1 | 0.5 ± 0.1 | 0.6 ± 0.2 | 0.7 ± 0.1 | 0.8 ± 0.0 |
| **15:0** | 0.2 ± 0.0 | 0.2 ± 0.0 | 0.2 ± 0.0 | 0.2 ± 0.0 | 0.2 ± 0.0 | 0.2 ± 0.0 | 0.2 ± 0.1 |
| **15:0**anteiso | - | 0.1 ± 0.1 | - | - | - | - | - |
| **15:0**iso | 0.1 ± 0.1 | - | - | 0.1 ± 0.0 | 0.1 ± 0.0 | 0.04 ± 0.1 | 0.1 ± 0.0 |
| **16:0** | 17.3 ± 3.3 | 16.1 ± 1.8 | 14.4 ± 1.9 | 14.4 ± 0.7 | 14.4 ± 1.2 | 16.0 ± 0.7 | 14.5 ± 0.5 |
| **16:1*n*-5** | 0.3 ± 0.0 | 0.4 ± 0.1 | 0.3 ± 0.0 | 0.4 ± 0.1 | 0.3 ± 0.0 | 0.3 ± 0.0 | 0.3 ± 0.1 |
| **16:1*n*-7** | 0.9 ± 0.2 | 1.0 ± 0.2 | 0.7 ± 0.2 | 0.7 ± 0.1 | 0.7 ± 0.1 | 1.1 ± 0.1 | 0.9 ± 0.2 |
| **16:1*n*-9** | 0.6 ± 0.1 | 0.8 ± 0.1 | 0.6 ± 0.2 | 0.5 ± 0.0 | 0.6 ± 0.1 | 0.7 ± 0.1 | 0.8 ± 0.0 |
| **16:2*n*-6** | 0.3 ± 0.1 | 0.5 ± 0.2 | 0.4 ± 0.1 | 0.3 ± 0.0 | 0.3 ± 0.1 | 0.4 ± 0.1 | 0.3 ± 0.1 |
| **16:3*n*-3** | 3.7 ± 0.1 | 4.9 ± 0.4 | 4.6 ± 1.2 | 4.4 ± 0.3 | 4.3 ± 0.6 | 4.4 ± 0.9 | 4.1 ± 0.8 |
| **16:4*n*-3** | - | - | 0.1 ± 0.0 | - | - | - | 0.1 ± 0.1 |
| **17:0** | 0.7 ± 0.1 | 0.6 ± 0.0 | 0.7 ± 0.1 | 0.6 ± 0.1 | 0.7 ± 0.1 | 0.6 ± 0.1 | 0.6 ± 0.1 |
| **17:0**anteiso | 1.1 ± 0.3 | 0.8 ± 0.2 | 0.9 ± 0.2 | 0.9 ± 0.1 | 0.9 ± 0.2 | 0.8 ± 0.1 | 0.9 ± 0.2 |
| **17:0**iso | 0.1 ± 0.0 | 0.2 ± 0.2 | 0.1 ± 0.1 | 0.1 ± 0.0 | 0.1 ± 0.0 | 0.1 ± 0.1 | 0.1 ± 0.0 |
| **17:1*n*-7** | 0.8 ± 0.3 | 0.5 ± 0.2 | 1.1 ± 0.2 | 0.7 ± 0.1 | 1.0 ± 0.3 | 0.8 ± 0.3 | 1.1 ± 0.2 |
| **18:0** | 4.6 ± 0.7 | 3.8 ± 0.2 | 3.9 ± 0.2 | 4.3 ± 0.6 | 4.5 ± 0.9 | 4.3 ± 0.7 | 4.0 ± 0.2 |
| **18:0**iso | - | 0.1 ± 0.0 | - | - | - | - | 0.1 ± 0.0 |
| **18:1*n*-11** | 0.5 ± 0.1 | 0.3 ± 0.0 | 0.4 ± 0.0 | 0.4 ± 0.0 | 0.5 ± 0.0 | 0.4 ± 0.1 | 0.7 ± 0.3 |
| **18:1*n*-7** | 0.8 ± 0.3 | 0.9 ± 0.2 | 0.6 ± 0.1 | 0.6 ± 0.1 | 0.6 ± 0.1 | 0.9 ± 0.1 | 0.9 ± 0.3 |
| **18:1*n*-9** | 9.4 ± 1.6 | 9.1 ± 0.6 | 7.3 ± 0.9 | 8.3 ± 0.4 | 8.2 ± 0.6 | 9.3 ± 0.7 | 9.5 ± 0.8 |
| **18:2*n*-3** | - | - | - | - | - | - | 0.1 ± 0.0 |
| **18:2*n*-6** | 2.9 ± 0.1 | 3.4 ± 0.5 | 3.2 ± 0.2 | 3.3 ± 0.3 | 3.2 ± 0.2 | 3.2 ± 0.1 | 2.7 ± 0.5 |
| **18:3*n*-3** | 13.7 ± 0.7 | 15.5 ± 0.6 | 14.7 ± 1.3 | 15.2 ± 1.7 | 14.2 ± 0.7 | 14.8 ± 1.4 | 13.5 ± 0.2 |
| **18:3*n*-6** | 0.6 ± 0.1 | 0.6 ± 0.0 | 0.5 ± 0.2 | 0.7 ± 0.1 | 0.6 ± 0.0 | 0.6 ± 0.1 | 0.5 ± 0.2 |
| **18:4*n*-3** | 0.5 ± 0.0 | 0.6 ± 0.1 | 0.5 ± 0.1 | 0.6 ± 0.0 | 0.5 ± 0.0 | 0.6 ± 0.1 | 0.5 ± 0.1 |
| **19:0** | 0.1 ± 0.0 | 0.1 ± 0.0 | 0.1 ± 0.0 | 0.1 ± 0.0 | 0.1 ± 0.0 | 0.1 ± 0.0 | 0.1 ± 0.1 |
| **19:0**iso | 0.1 ± 0.0 | 0.1 ± 0.0 | - | 0.1 ± 0.0 | - | 0.1 ± 0.0 | 0.1 ± 0.0 |
| **19:1*n*-9** | 0.2 ± 0.0 | 0.2 ± 0.0 | 0.1 ± 0.0 | 0.1 ± 0.0 | 0.1 ± 0.0 | 0.1 ± 0.0 | 0.2 ± 0.0 |
| **20:0** | 0.5 ± 0.1 | 0.4 ± 0.1 | 0.3 ± 0.1 | 0.3 ± 0.0 | 0.3 ± 0.0 | 0.5 ± 0.1 | 0.4 ± 0.1 |
| **20:1*n-*11** | 2.0 ± 0.2 | 1.8 ± 0.3 | 2.1 ± 0.3 | 2.1 ± 0.2 | 2.2 ± 0.2 | 2.2 ± 0.4 | 2.3 ± 0.0 |
| **20:1*n*-7** | 0.1 ± 0.0 | 0.2 ± 0.0 | 0.1 ± 0.0 | 0.1 ± 0.0 | 0.1 ± 0.0 | 0.1 ± 0.0 | 0.2 ± 0.1 |
| **20:1*n*-9** | 2.2 ± 0.4 | 2.3 ± 0.2 | 1.9 ± 0.3 | 1.9 ± 0.4 | 1.8 ± 0.2 | 2.1 ± 0.3 | 2.1 ± 0.2 |
| **20:2*n*-6** | 1.6 ± 0.2 | 1.8 ± 0.1 | 1.9 ± 0.1 | 1.9 ± 0.2 | 2.0 ± 0.2 | 1.9 ± 0.4 | 1.8 ± 0.1 |
| **20:2*n*-9** | 1.9 ± 0.4 | 1.6 ± 0.2 | 1.9 ± 0.4 | 1.9 ± 0.1 | 1.7 ± 0.1 | 1.6 ± 0.1 | 2.4 ± 0.5 |
| **20:3*n*-3** | 2.8 ± 0.6 | 3.3 ± 0.4 | 3.5 ± 0.5 | 3.2 ± 0.5 | 3.5 ± 0.1 | 3.2 ± 0.6 | 3.6 ± 0.3 |
| **20:3*n*-6** | 1.0 ± 0.2 | 1.3 ± 0.3 | 1.1 ± 0.2 | 1.0 ± 0.3 | 0.9 ± 0.1 | 1.1 ± 0.2 | 1.0 ± 0.1 |
| **20:3*n*-7** | 0.2 ± 0.0 | 0.2 ± 0.0 | 0.3 ± 0.0 | 0.3 ± 0.0 | 0.3 ± 0.1 | 0.3 ± 0.0 | 0.3 ± 0.0 |
| **20:3*n*-9** | 0.1 ± 0.0 | 0.1 ± 0.0 | 0.1 ± 0.0 | - | - | 0.1 ± 0.0 | 0.1 ± 0.0 |
| **20:4*n*-3** | 0.9 ± 0.2 | 1.3 ± 0.4 | 0.9 ± 0.2 | 0.7 ± 0.3 | 0.6 ± 0.0 | 1.0 ± 0.3 | 0.9 ± 0.2 |
| **20:4*n*-6** | 8.4 ± 0.6 | 7.5 ± 0.9 | 9.5 ± 0.4 | 10.0 ± 1.4 | 9.9 ± 0.6 | 7.7 ± 1.1 | 8.0 ± 0.9 |
| **20:5*n*-3** | 6.7 ± 1.0 | 7.0 ± 0.7 | 7.7 ± 1.0 | 7.0 ± 0.4 | 7.6 ± 0.3 | 7.2 ± 0.6 | 7.5 ± 0.4 |
| **21:0** | - | - | - | - | - | - | - |
| **22:0** | 0.4 ± 0.1 | 0.3 ± 0.1 | 0.3 ± 0.1 | 0.2 ± 0.0 | 0.2 ± 0.1 | 0.3 ± 0.1 | 0.3 ± 0.0 |
| **22:1*n*-11** | 0.3 ± 0.0 | 0.2 ± 0.0 | 0.2 ± 0.0 | 0.1 ± 0.1 | 0.3 ± 0.2 | 0.3 ± 0.1 | 0.2 ± 0.0 |
| **22:1*n-*9** | 0.2 ± 0.1 | 0.1 ± 0.0 | 0.1 ± 0.0 | - | 0.1 ± 0.1 | 0.1 ± 0.0 | 0.1 ± 0.1 |
| **22:2*n*-6** | 0.1 ± 0.0 | 0.1 ± 0.0 | 0.1 ± 0.0 | 0.6 ± 0.9 | 0.6 ± 0.9 | 0.1 ± 0.0 | 0.1 ± 0.0 |
| **22:2*n*-9** | 1.7 ± 0.4 | 1.7 ± 0.3 | 1.6 ± 0.4 | 1.2 ± 1.0 | 1.1 ± 1.0 | 1.8 ± 0.3 | 1.9 ± 0.1 |
| **22:3*n*-6** | 0.3 ± 0.0 | 0.3 ± 0.1 | 0.5 ± 0.1 | 0.4 ± 0.1 | 0.4 ± 0.1 | 0.4 ± 0.2 | 0.4 ± 0.1 |
| **22:4*n*-6** | 5.3 ± 1.3 | 4.3 ± 0.8 | 6.3 ± 0.9 | 6.9 ± 0.5 | 6.2 ± 0.1 | 4.5 ± 0.6 | 5.8 ± 0.5 |
| **22:5*n*-3** | 2.6 ± 0.6 | 2.7 ± 0.2 | 3.1 ± 0.8 | 2.4 ± 0.2 | 2.8 ± 0.5 | 3.2 ± 0.6 | 3.0 ± 0.2 |
| **24:0** | 0.1 ± 0.0 | 0.1 ± 0.0 | 0.1 ± 0.0 | 0.1 ± 0.0 | - | 0.1 ± 0.0 | 0.1 ± 0.0 |
